# L'Hopital Du Gros-Caillou

**Published:** 1831-02

**Authors:** 


### L'HOPITAL DU GROS-CAILLOU.

### *On the Propriety of performing the Operation of Amputation immediately after the Receipt of Injury.*

We believe that the experience of our own and foreign military surgeons, during the late Peninsular war, has almost entirely overcome any doubt that previously existed upon this most important question. Mr. Guthrie* remarks, in the preface to his valuable work, that, "before the termination of the war in 1815, and the appearance of the first edition of this work, the opinions of Mr. Hunter on the powers and capabilities of the human constitution were universally received. As general principles, they did little mischief; but when they came to be acted upon, the results were not found to coincide with the principles from which they were deduced. When an injury had occurred to a person in health, rendering the loss of a limb necessary, he recommended that an operation should not be performed until after suppuration had been established, a period of probably six weeks, which, even if the patient survived, was often found too late to be serviceable. From the failure of this practice, the contrary one, of immediate amputation, became gradually more general during the war, and at its close, I not only advocated and established the propriety of it, but examined the reasoning on which Mr. Hunter's opinions were founded, and, I trust, have proved it to be defective."

At page 231 of the same work, Mr. Guthrie also states, that "when the surgeon is satisfied there is no chance of saving the limb by prudent delay, the operation is to be performed as soon after the receipt of the injury as the state of the patient will permit: the only point to be considered is, if the patient has so far recovered the shock of the injury as to be able to bear the additional one of the operation."

Baron Larrey has greatly contributed, by his practice and writings, to prove the advantages of amputation soon after the infliction of injury; and the memorable conflict of July last, in the streets of Paris, afforded him additional opportunities of proving the solidity of his doctrine. He states, in his "Surgical Account of the last Days of July," which was read at the Academy of Sciences, that several cases occurred at his hospital, which tended completely to verify the "hitherto undecided" question respecting the period when the amputation of a limb should be performed. All the wounded who suffered amputation during the first twenty-four hours were perfectly cured, or their cure was much advanced, and the cicatrization of the wounds had not been interrupted by any serious accident. Of this number were five young guardsmen, upon whom amputation of the arm or thigh was performed by

* Treatise on Gunshot Wounds, &c. Third edition.

MM. Barthelemi and H. Larrey, the Baron's son. The stumps were healed by the first intention. The consecutive amputations, on the contrary, were generally followed by great disturbance, which was, however, relieved in most instances. In some, local symptoms only occurred; as tetanic spasms, passive hemorrhage, erysipelas, traumatic and hospital gangrene. In others, the constitution suffered considerably, and the internal organs were sympathetically affected. Secondary hemorrhage was prevented by the application of ice to the stumps. The traumatic erysipelas was remedied by red-hot irons applied to each inflamed part. The same means, together with mild emetics, rapidly arrested the hospital gangrene, which invaded the wounds of some of the patients who had been operated upon.

Baron Larrey also obtained additional evidence of the advantage of the practice of amputating a limb, during the existence of traumatic gangrene, without waiting for a line of separation between the diseased and sound parts. In the consecutive amputations, the edges of the wound were brought lightly together, and no endeavour was made to stretch the parts, to bring them into exact contact: this practice, in the Baron's opinion, tended greatly to ensure the safety of the patients.

